# Cervical cancer screening in Australia: modelled evaluation of the impact of changing the recommended interval from two to three years

**DOI:** 10.1186/1471-2458-10-734

**Published:** 2010-11-26

**Authors:** Prudence Creighton, Jie-Bin Lew, Mark Clements, Megan Smith, Kirsten Howard, Suzanne Dyer, Sarah Lord, Karen Canfell

**Affiliations:** 1 Cancer Epidemiology Research Unit, Cancer Council NSW, Sydney, Australia; 2 National Centre for Epidemiology and Population Health, Australian National University, Canberra, Australia; 3 Screening and Test Evaluation Program, School of Public Health, University of Sydney, Australia; 4 NHMRC Clinical Trials Centre, University of Sydney, Sydney, Australia; 5 School of Public Health, University of Sydney, Australia; 6 School of Public Health and Community Medicine, University of NSW, Sydney, Australia

## Abstract

**Background:**

The National Cervical Screening Program in Australia currently recommends that sexually active women between the ages of 18-70 years attend routine screening every 2 years. The publically funded National HPV Vaccination Program commenced in 2007, with catch-up in females aged 12-26 years conducted until 2009; and this may prompt consideration of whether the screening interval and other aspects of the organized screening program could be reviewed. The aim of the current evaluation was to assess the epidemiologic outcomes and cost implications of changing the recommended screening interval in Australia to 3 years.

**Methods:**

We used a modelling approach to evaluate the effects of moving to a 3-yearly recommended screening interval. We used data from the Victorian Cervical Cytology Registry over the period 1997-2007 to model compliance with routine screening under current practice, and registry data from other countries with 3-yearly recommendations to inform assumptions about future screening behaviour under two alternative systems for screening organisation - retention of a reminder-based system (as in New Zealand), or a move to a call-and-recall system (as in England).

**Results:**

A 3-yearly recommendation is predicted to be of similar effectiveness to the current 2-yearly recommendation, resulting in no substantial change to the total number of incident cervical cancer cases or cancer deaths, or to the estimated 0.68% average cumulative lifetime risk of cervical cancer in unvaccinated Australian women. However, a 3-yearly screening policy would be associated with decreases in the annual number of colposcopy and biopsy procedures performed (by 4-10%) and decreases in the number of treatments for pre-invasive lesions (by 2-4%). The magnitude of the decrease in the number of diagnostic procedures and treatments would depend on the method of screening organization, with call-and-recall screening associated with the highest reductions. The cost savings are predicted to be of the order of A$10-18 M annually, equivalent to 6-11% of the total cost of the current program (excluding overheads), with call-and-recall being associated with the greatest savings.

**Conclusions:**

Lengthening the recommended screening interval to 3 years in Australia is not predicted to result in increases in rates of cervical cancer and is predicted to decrease the number of women undergoing diagnostic and treatment procedures. These findings are consistent with a large body of international evidence showing that screening more frequently than every three years with cervical cytology does not result in substantial gains in screening effectiveness.

## Background

The National Cervical Screening Program in Australia was introduced in 1991, and has been very successful in reducing the incidence and mortality from cervical cancer. The program recommends 2-yearly screening for sexually active women aged from 18-20 to 69 years [1]. This interval is shorter than that suggested by the International Agency for Research on Cancer (IARC), which recommends a 3-yearly screening interval for women aged 25-49 and a 5-yearly interval for women aged 50-65 years [2]. When trends in cervical cancer incidence and mortality in Australia were compared with those in England, similar reductions were observed after the introduction of organised screening in each country, despite the recommended 3-yearly interval that was in place in many regions in England prior to 2003; demonstrating that the 2-yearly policy in Australia has been of broadly similar effectiveness to a predominantly 3-yearly policy in the UK [3].

Australia initiated a national human papillomavirus (HPV) public vaccination program in 2007, and this included a two year catch-up phase for females aged 12-26 years. Although final coverage estimates for the GP-based component of the catch-up program in older females have not yet been released, early indications are that coverage rates in this group are likely to be relatively high [4,5]. Ecological data already indicate a falling incidence in genital warts in young Australian females, which is likely to be due to roll-out of a quadrivalent vaccine that also protects against HPV types 6 and 11, which are implicated in the development of genital warts [4,5]. As the vaccinated cohorts mature, cervical screening will inevitably become less cost-effective over time, because the average risk of cervical cancer in the Australian population will eventually fall due to vaccination. In this context, measures to increase the efficiency of screening are of interest. In the current evaluation we took a near term approach and did not explicitly incorporate the effect of vaccination on the cost-effectiveness of 3-yearly screening; rather, the changes in cancer outcomes and cost-effectiveness associated with a move to 3-yearly screening in the current evaluation should be considered "worst case" from a safety perspective (since vaccination will eventually reduce the risk of invasive cervical cancer in the population).

The National Cervical Screening Program in Australia uses a reminder-based system, in which women who are registered on state or territory cytology registries are sent a letter if they do not have a screening test at the recommended interval. We have previously suggested that if the recommended screening interval is increased, a move to a call-and-recall system (in which proactive invitations to attend screening are issued) could be considered in order to increase compliance with the new recommendation and to limit late re-screening [6]. The use of a call-and-recall system in England, and other differences in the organisation of screening, have been associated with a higher degree of compliance with the recommended interval in England compared to Australia [3].

The objective of the current evaluation was to assess the epidemiologic outcomes and cost-effectiveness implications of changing the recommended screening interval in Australia to 3 years, under two alternative assumptions about screening organisation - retention of the current reminder-based system, or a move to call-and-recall organisation. Specifically, the aims of the study were to quantify the predicted effect of a 3-yearly screening recommendation in Australia on: (1) national rates of invasive cervical cancer cases and cancer deaths; (2) the estimated average cumulative lifetime risk of cervical cancer in Australian women; (3) the annual number of colposcopy and biopsy procedures nationally; (4) the number of treatments for pre-invasive lesions nationally; and (5) costs and cost-effectiveness. We used screening registry data from the UK to inform compliance assumptions in Australia if a move to a 3-yearly recommendation was also associated with a move to call-and-recall organisation. Because the New Zealand (NZ) national cervical screening program recommends a 3-yearly interval but uses a reminder-based system, we used screening registry data from NZ to inform 3-yearly compliance assumptions if the reminder-based system was retained in Australia.

## Methods

### Model construction

**Figure 1:**
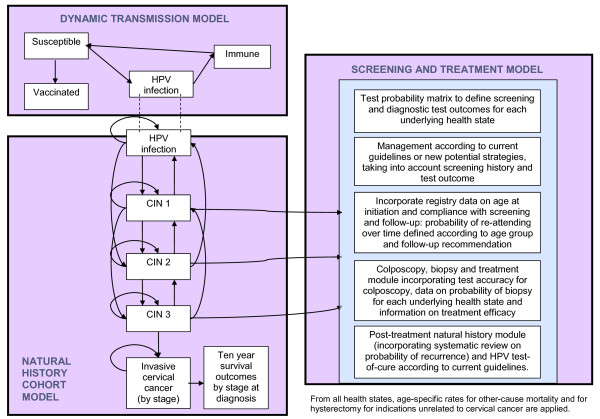
**Schematic diagram of model structure**.

A schematic diagram of the model is provided in Figure 1; details of model structure, calibration and validation have been described elsewhere [6-10]. Briefly, cervical screening was simulated using a discrete time, deterministic Markov cohort model of the natural history of oncogenic HPV infection, cervical intraepithelial neoplasia (CIN) and cervical cancer which was integrated with a detailed decision tree of screening according to current management and treatment guidelines in Australia [1]. The simulation follows a cohort of women from ages 10 to 84. The age-specific incidence of HPV was obtained from a dynamic model of sexual behaviour and HPV transmission in Australia [10], and for this study it was assumed that the HPV incidence reflected an unvaccinated cohort.

Screening structures and parameters included: detailed characterisation of the age distribution of starting screening in the population (screening initiation) and age-specific compliance with current screening and all specific management recommendations using registry information; the results of a systematic review on cytology test accuracy [8], analysis of biopsy rates and colposcopy-histology correlation rates from a large database involving over 21,000 examinations conducted between 1998 and 2007 at the Royal Women's Hospital, Melbourne (described in [11,12]); treatment compliance and efficacy, post-treatment management and test-of-cure; stage-specific cervical cancer survival using registry data from the state of New South Wales (NSW); hysterectomy rates; and costs.

The natural history model of CIN and invasive cervical cancer was updated from an earlier implementation [7]. Feasible ranges for natural history transitions were assessed via literature review and the baseline values were then calibrated to reproduce cervical cancer incidence in unscreened populations. When complete screening and management structures were included, the model was calibrated to reproduce age-specific Australian data on HPV prevalence, screen-detected and histologically-confirmed high grade CIN, and cancer incidence and mortality rates [8,9] (subsequently, some parameter changes were made to accommodate the further calibration target of the age-specific stage distribution of cervical cancer at diagnosis; this did not have a substantial effect on the predicted lifetime costs and effects). When the age-specific event rates predicted by the model of current screening were applied to the 2007 Australian population, model predictions were confirmed to broadly concur with recent observed data for the predicted observed annual numbers of incident cervical cancer cases and cancer deaths and the number of cytological smears occurring annually in the cervical screening program (including all smears taken for screening, follow-up and as part of subsequent pre-and post-treatment management - ~2 million smears). The total annual costs of the cervical screening program, including all follow-up and diagnostic tests and the costs of managing invasive cervical cancer (but excluding overheads) was estimated as approximately $A166 M [8,9].

Under current management guidelines in Australia, women with high grade cytological results are directly referred to colposcopy but women with possible and definite low grade lesions (known as pLSIL and dLSIL in the Australian Modified Bethesda System and broadly equivalent to ASCUS and LSIL in The Bethesda System 2001) are followed up at 12 months with repeat cytology, unless the women is over 30 years of age without a recent history of normal cytology. In our evaluation these follow-up visits were explicitly modelled to take into account any progression or regression of disease during the follow-up period. The systematic review of cytology test accuracy, in conjunction with the observed laboratory "call" rates for low and high grade lesions in Australia, was used to estimate the point on the cytology receiver operating curve at which laboratories in Australia are operating (as described in [8]). This information was then used to construct a test probability matrix which described the relationship between each underlying natural history health state in the model and the probability of each possible cytology test result. The matrix completely describes the assumed test accuracy. As an example, the baseline cross-sectional sensitivity described by the matrix for histologically confirmed high-grade disease (CIN2+) at a cytology low grade (dLSIL) threshold is 72%. However, it should be noted that this does not describe the effective "operational" sensitivity of cytology in Australia, which depends on the specific management guidelines and referral processes that we modelled.

The model was implemented in TreeAge Pro 2008 (Release 1.3.2, TreeAge Software, Inc., MA, USA).

### Analysis of screening registry data

The baseline model of cervical screening in Australia incorporated the results of an analysis of approximately 6.3 million satisfactory smears from the Victorian Cervical Cytology Register from the period 1 Jan 1997-31 Dec 2007. The state of Victoria has about one third of Australia's population, and the state's cervical cytology registry was the first to be established in Australia. Using the Victorian data, age-specific re-screening probabilities over time were derived from analysis of data from ~1.7 million women with a negative smear. Re-screening probabilities over time were calculated and stratified by the previous cytological smear result and the recommended recall timing after the previous test. Interval-specific re-screening probabilities were calculated for 10 year age-groups, over a period of 10 years after the index test. For women who did not re-screen within 10 years of their last test, annual interval-specific re-screening probabilities at 10+ years were assumed to vary with age, decreasing from 30% of those who had not yet been re-screened (in the age group 15-39 years) to 5% (ages 70 years or more), to reflect the lower probability of these underscreened women re-attending for screening at ages older than the target age group for screening. Because the degree of loss to follow-up over period of longer than 10 years is uncertain, these probabilities were varied in sensitivity analysis.

The Victorian registry data provided follow-up dates by month and year, but we categorised the follow-up duration according to the time step used by the model; which is 12 months, except at specific points where natural history and screening transitions were adjusted to reflect a 6 month time step to model specific follow-up pathways as required by the Australian cervical screening guidelines [1]. For the registry data analysis, we defined the probability of re-attending for screening in the first annual time step as attendance from 0-15 months after the index test; the second annual time step included attendance from 16-30 months after the index test; and annual time steps thereafter included attendance during consecutive 12 month intervals (31-42 months; 43-54 months, etc.). For analysis of compliance data in situations where there was a recommended 6 month follow-up interval, the probability of re-attendance at 6 months for modelling purposes was calculated over the period 0-9 months after the index test.

In order to calculate the re-screening probabilities for hypothetical 3-yearly screening scenarios in Australia, we used previously published results for analysis of historical registry data on 3-yearly screening compliance from England in 2,497 women who had negative smears over the period 1988-1996 (Oxfordshire) [3] to inform compliance assumptions given a call-and-recall method of screening organisation; and analysis of data from ~1.1 million women in NZ who had 3.4 million negative smears over the period July 1 1995- June 30 2005 to inform compliance assumptions given a reminder-based system of organisation. Similar methods for calculating interval-specific probabilities of re-attending for screening were applied to the Oxfordshire and NZ registry data as were used for the Victoria data. However, because we did not have age-specific data for Oxfordshire, we scaled the all-ages re-screening probabilities over time according to age-specific coverage data for England in 1996 [13] in order to estimate age-specific re-screening probabilities.

We could not directly apply these re-screening probabilities from other countries to Australia in order to evaluate the differential effects of implementing 2 and 3-yearly screening recommendations, because the cumulative proportion re-screened over longer periods (at 5 years and at 10 years) differed in each country. If the data from other countries had been directly applied in the evaluation, the results would partly reflect country-specific differences in the behaviour of underscreened women who had not attended screening for 5 years or more. Therefore, for the main evaluation we adjusted the patterns of re-screening behaviour from other countries so that the cumulative proportion re-screened at 4 years and beyond was the same as currently observed in Australia. This was done so that any differences in outcomes between the screening strategies would reflect differences in re-screening before 4 years (which was assumed to be the period over which the main effects on screening behaviour of a change from a 2-yearly to a 3-yearly recommendation would be observed), rather than differences that occur between settings in re-screening patterns over longer periods of follow-up. Additionally, because NZ has a slightly higher level of early rescreening in the first year compared to Australia, and since we would not expect lengthening the recommended interval in Australia to be associated with increased levels of early re-screening in the first year, we also adjusted the re-screening probabilities from NZ so that re-screening behaviour in the first year was the same as currently observed in Australia. We then populated the model with the re-screening probabilities derived from the England and NZ data to simulate the two different 3-yearly screening strategies in Australian context.

As a secondary analysis we also assessed the cost-effectiveness of screening if compliance were exactly as observed in the other countries, even in underscreened women. We also explored the effects of different assumptions about early re-screening rates on the cost-effectiveness outcomes.

In the evaluation we assumed that the current Australian screening and management guidelines, and compliance to these guidelines, would remain unchanged in every respect other than in relation to routine screening behaviour in women with normal (negative) cytology results. For both call-and-recall and reminder-based systems we assumed, according to standard practice, that a women who re-screens early and receives a normal screening result is sent her next invitation and/or reminder letter after the usual recommended interval has elapsed following the early re-screening event.

### Outcomes evaluation

The outputs of the model included age-specific predicted rates of cytology test utilisation, colposcopies, biopsies and treatments for high grade CIN, cervical cancer incident cases and cancer deaths. Age-standardised rates of cancer incidence and mortality were calculated using the 2001 Australian standard population. The 2007 Australian female resident population was then applied to the age-specific rates to estimate annual resource utilisation, including annual numbers of screening tests, colposcopies, biopsies and treatments for high grade CIN; and case numbers of incident cervical cancer and of cancer deaths.

### Cost-effectiveness evaluation

We took a health services perspective, considering screening, diagnosis and treatment costs that would be billed to the Australian Medicare (government-reimbursed) system, in accordance with previous evaluations of cervical screening in Australia [8,9]. The model was populated with cost data from the Medicare Benefits Schedule for screening, diagnostic, and high grade CIN treatment procedures and the costs of invasive cervical cancer management; [8,9] with the reimbursed cost of a cytology smear currently being A$19.60. Lifetime costs and benefits were estimated for each scenario using standard cohort model methods. In accordance with standard practice for health economic evaluation of the incremental costs and effects of new strategies, we modelled the effect of different screening strategies on a specific cohort of women; rather than performing multiple cohort evaluation of the effect of changing strategies for older cohorts within the screening program who have been previously screened. The calculation of costs and life years commenced from age 18 (the earliest age at which screening is recommended to start in Australia) with discounting at a rate of 5%. Because the evaluation found that the strategies under consideration had close-to-equivalent effects but were less expensive than current practice, the calculation of incremental cost-effectiveness ratios (ICERs) was not meaningful or appropriate (when the effects of two strategies are almost equivalent, the calculated ICER approaches infinity); and therefore we presented incremental costs and health outcomes compared to current practice in a disaggregated form.

### Sensitivity analysis

Sensitivity analysis was performed to assess the robustness of the findings to the natural history and screening model parameters. We used a combination of one-way, and where appropriate, multi-way sensitivity approaches to analyse predicted effects and costs when subsets of parameters were varied. For the natural history sensitivity analysis, we applied alternate natural history parameter sets for CIN progression and regression probabilities, non-symptomatic invasive cancer progression probabilities and also specifically investigated the sensitivity of results to doubling and halving the parameters describing HPV incidence and progression of CIN 3 to localised invasive cervical cancer. For the screening parameters, one-way sensitivity analysis was performed to assess the sensitivity of results to increased or decreased early re-screening, age at screening initiation, the rate of cytology unsatisfactory results, colposcopy test accuracy and attendance at colposcopy. Sensitivity of the results to the assumed test characteristics of cytology was analysed using a multi-way approach to apply different parameter sets which described the probability of a particular cytology test result given an underlying health state; these multiple parameter sets were derived following systematic review of the literature on cytology test accuracy and are described in detail in previous work [8]. Secondary analysis was also performed to assess sensitivity of predicted effects and costs to long term coverage parameters (as informed by the analysis of NZ and UK re-screening data) and the re-screening probabilities after 10 years since the last screening test.

### Ethical approval

The new screening registry data and other sources of new data used in the model were deidentified, and the Cancer Council NSW Human Research Ethics Committee approved the transfer of these datasets to the researchers and their use in modelled evaluation.

## Results

### Analysis of screening registry data

**Figure 2:**
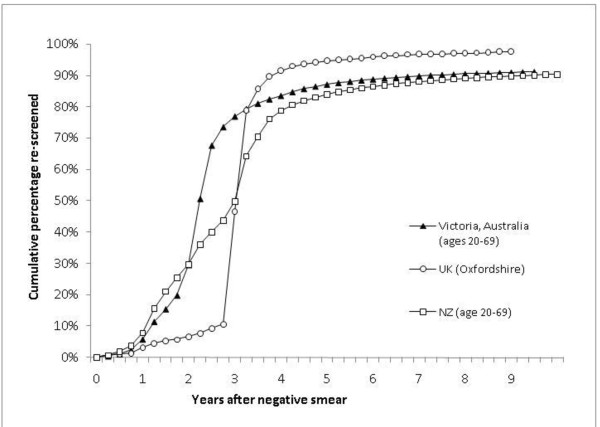
**Cumulative proportion of women with a negative smear re-screened over time in Australia, NZ and the UK**. Australian data are for 1.7 million women listed on the Victorian Cervical Cytology Registry who had a negative smear between 1997 and 2007. NZ data are for 1.1 million women listed on the NZ National Cervical Screening Registry who had a negative smear between 1995 and 2005. UK data are for 2,497 randomly selected Oxfordshire participants in the Million Women Study who had a negative smear between 1988 and 1996 [3].

The cumulative proportions of women with a negative smear who were re-screened by time since the index smear for the three countries are shown in Figure 2. Overall, the data from Oxfordshire show a high level of compliance to a recommended 3-yearly interval in the context of call-and-recall organisation. Compared to Oxfordshire, data from NZ show a greater level of earlier re-screening in the context of a 3-yearly recommendation; but the median interval at which women were re-screened was similar (36 months for NZ and 38 months for Oxfordshire). As expected, the median interval at which women are screened in Victoria, Australia is currently shorter than in the other two countries, at 27 months, reflecting the current 2-yearly screening recommendation. The finding for Victoria is consistent with prior work on screening compliance in Australia showing a median re-screening interval of 27 months in NSW women with an index normal screening test in 1998 [3].

For the main cost-effectiveness evaluation of 3-yearly screening in Australia, and based on the data from the UK and NZ shown in Figure 2, we assumed that if the current reminder-based system was retained, there would be approximately 44% early re-screening before 33 months, whereas if a call-and-recall system was implemented, there would be 11% early rescreening before 33 months.

### Outcomes evaluation

Table 1 shows the observed case numbers and rates of cervical cancer and cervical cancer deaths in Australia, compared to baseline model predictions for current practice using the screening data from Victoria to model current levels of compliance to screening and management recommendations (baseline predictions were calibrated to the observed age-specific rates [8,9]). The model predicts no substantial change in the average cumulative lifetime risk of cervical cancer in Australian women (~0.68%) if 3-yearly screening were to be implemented under either a reminder-based or call-and-recall system. A total of 736 incident cases are predicted annually for 2-yearly screening; 732 for 3-yearly screening with a reminder-based system; and 731 for 3-yearly screening with call-and-recall organisation. A total of 208 cervical cancer deaths are predicted annually for 2-yearly screening; 205 for 3-yearly screening with a reminder-based system; and 204 for 3-yearly screening with call-and-recall organisation. Therefore, the number of cancers and cancer deaths were broadly similar for all scenarios, with the magnitude of the predicted differences between strategies being less than the relatively small difference between baseline predictions for cancer incidence and mortality and the observed data. Because of the counter-intuitive baseline finding that 3-yearly screening had very slightly improved outcomes than 2-yearly screening, we performed further investigation of the reason for this effect, finding that it was due to complex issues of modelling the timing of re-screening in women still not screened after 4 years, in repeated rounds of screening. We also performed extensive sensitivity analysis to characterise this effect under different assumptions about late re-screening, as described in detail in the *Sensitivity Analysis *section of our results.

**Table 1:** Observed and predicted health outcomes in Australia

	**Cervical cancer cases**^**(1)**^	**Cervical cancer incidence per 100,000**^**(2)**^	**CLR**^**(3) **^**cervical cancer**	**Cervical cancer deaths**^**(1)**^	**Cervical cancer mortality per 100,000**^**(2) **^
**Observed**^(4)^	723	6.8	--	219	1.9

**Current practice**, **2-yearly recommendation**	736	6.8	0.68%	208	1.9

**Continue reminder system**, **3-yearly recommendation**	732	6.7	0.67%	205	1.8

**Call-and-recall**, **3-yearly recommendation**	731	6.7	0.67%	204	1.8

Notes

(1) Model-predicted cases and deaths in women aged 0-84 years are estimated using the resident Australian female population in 2007.

(2) Age-standardised to the 2001 Australian population.

(3) CLR = cumulative lifetime risk

(4) Average 2004-2006 [21]

Table 2 shows the predicted healthcare utilisation implications of the new screening strategies. Compared to current practice, a move to a 3-yearly recommendation would reduce the annual number of cervical smears, and also reduce the number of colposcopies, biopsies and treatments occurring within the program. The reductions would be greater for the call-and-recall strategy, due to increased compliance to the 3-yearly interval and consequently lower levels of early re-screening. The model predicts that a move to 3-yearly screening in Australia would reduce the annual numbers of cytology tests by 7-13%, depending on whether call-or-recall or reminder-based organisation was used (with the reduction ranging from 7-18% in sensitivity analysis). The number of colposcopies and biopsies would be reduced by 4-10% (ranging from 3-13% in sensitivity analysis), and the number of treatments would be reduced by 2-4% (ranging from 1-6% in sensitivity analysis). In the baseline model, this is equivalent to between 140,000-250,000 fewer cytology tests, between 2,700-6,400 fewer colposcopies, between 1,400-3,200 fewer biopsies, and between 300-600 fewer treatments for high grade CIN2/3.

**Table 2:** Predicted health resources utilisation in Australia(1)

	Total cytology tests (and change from current practice)	Total colposcopies (and change from current practice)	Total biopsies (and change from current practice)	Total CIN 2/3 treatments (and change from current practice)
Current practice, 2-yearly recommendation	1.90M	66,000	32,000	17,600

Continue reminder system, 3-yearly recommendation	1.76M (140,000 fewer)	63,300 (2,700 fewer)	30,600 (1,400 fewer)	17,300 (300 fewer)

Call-and-recall, 3-yearly recommendation	1.65M (250,000 fewer)	59,600 (6,400 fewer)	28,800 (3,200 fewer)	17,000 (600 fewer)

Note: (1) Estimated for women of all ages using the resident Australian female population in 2007.

### Cost-effectiveness evaluation

**Figure 3:**
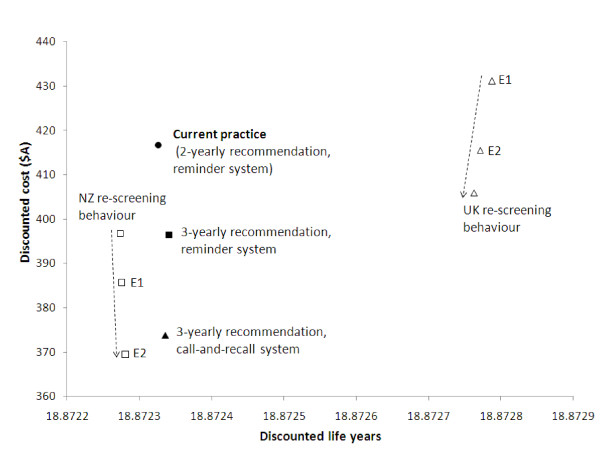
**Cost-effectiveness of current practice with 2-yearly screening and of potential 3-yearly screening strategies in Australia**. The positions on the cost-effectiveness plane of the main screening strategies for the evaluation are indicated with solid symbols. These strategies had equivalent compliance in underscreened women (equivalent levels of re-attendance after 4 years from the last screening test were assumed). Open symbols indicate position on the cost-effectiveness plane if screening compliance was exactly as observed in NZ and the UK, even at periods greater than 4 years. Strategies marked E1 and E2 involve alternate assumptions about early re-screening - E1 indicates that early re-screening levels at 12 months are equivalent to those currently observed in Australia, whereas E2 indicates that this level of rescreening is observed over 24 months (dotted arrows indicate decreasing levels of early re-screening).

Figure 3 shows the position on the cost-effectiveness plane of the screening strategies evaluated. Compared to current practice, the main 3-yearly screening strategies under consideration are associated with close-to-equivalent effectiveness in terms of life years saved by cervical cancer screening, consistent with the findings reported in Table 1 that the 3-yearly screening strategies are not expected to increase the number of cervical cancer cases or deaths. Table 3 shows the costs and effectiveness implications of the main strategies.

**Table 3:** Estimated total annual costs and effects of cervical screening in Australia, excluding overheads (all costs in Australian dollars)

	Annual costs to health care system	Direct cytology cost component	Discounted lifetime cost *(5% discount rate)*	Discounted life-years *(5% discount rate)*	Cost-effectiveness ratio compared to no intervention
**No intervention**	$34.7M*	-	$52.1*	18.8619	-

**Current practice**, 2-yearly recommendation	$166.7M	$37.4 M (22%)	$416.7	18.8723	$35,000/LYS

**Continue reminder system**, 3-yearly recommendation	$156.7M	$34.4 M (22%)	$396.4	18.8723	$33,000/LYS

**Call-and-recall**, 3-yearly recommendation	$148.8M	$32.3 (22%)	$373.8	18.8723	$31,000/LYS

*Estimated cost of cervical cancer treatment in an unscreened population

As shown in Figure 3 and Table 3, the 3-yearly screening strategies would be associated with a reduction in costs to the health care system, with no substantial change in health outcomes, thereby presenting better value for money than the current program. A substantial component of the cost saving would result from the reduction in direct costs of cytology testing (Table 3), but savings would also result from an overall reduction in the numbers of diagnostic and treatment procedures. The cost savings are predicted to be of the order of A$10 M -$18 M annually, equivalent to 6-11% (range ~5-15% in sensitivity analysis) of the total cost of the current program, depending on the method of screening organisation, with call-and-recall being associated with the greatest savings (although any additional overhead costs associated with call-and-recall are not considered in the current evaluation). Under all strategies, direct cytology costs would be associated with approximately 22% of the overall costs of the program (not including overheads). As previously discussed, ICERs for the new strategies vs. current practice would not be meaningful due to their very similar effectiveness. However, Table 3 gives an estimate of the cost-effectiveness ratio of each strategy compared to no screening intervention (in which the costs involved for the hypothetical strategy of no screening would result from treatment of invasive cervical cancer). This suggests that the current screening program is cost-effective, with a cost-effectiveness ratio compared to no intervention of $35,000 per life year saved, but also that cost-effectiveness would be improved if the 3-yearly screening strategies were implemented.

Figure 3 also shows the cost-effectiveness findings for the supplementary 3-yearly screening strategies that were evaluated in secondary analysis. These supplementary evaluations assumed that screening compliance was exactly as observed in the reference countries of NZ and the UK, even for women who had not re-screened for 4 years or more after an index negative smear. Because the data from Oxfordshire show very high levels of re-screening by 5 years (which is likely related to practitioner incentives structure around 5-yearly coverage rates in the UK [3]), the overall effectiveness of screening is increased compared to the other strategies evaluated. Figure 3 also shows the effect of alternate assumptions about early rescreening in context of the UK and NZ re-screening behaviour. As expected, as early rescreening before 3 years decreases, the costs of the screening program decrease but there is very little impact on effectiveness.

### Sensitivity analysis

**Figure 4:**
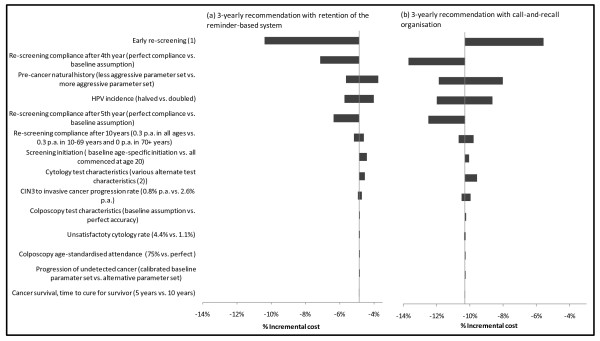
**Sensitivity analysis for incremental costs of 3-yearly screening vs. current practice with a 2-yearly recommendation**. *Note: *(1) When assumptions about early re-screening in both 2 and 3-yearly screening scenarios were varied, the direction of the incremental effect on 3-yearly screening was different for the two different 3-yearly screening scenarios. For a move to a 3-yearly recommendation with retention of the reminder-based system, a higher early re-screening rate resulted in an increase in the cost savings; for a move to 3-yearly screening with call-and-recall organisation a higher early re-screening rate decreased the cost-savings. (2) When cytology test characteristics in 2 and 3-yearly screening scenarios were varied, the direction of the incremental effect on 3-yearly screening was different for the two different 3-yearly screening scenarios.

**Figure 5:**
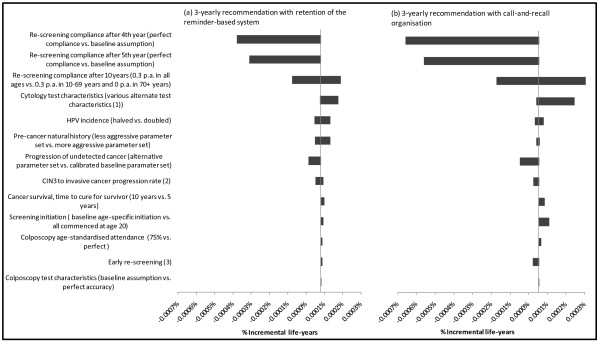
**Sensitivity analysis for incremental effect on life years saved or lost with 3-yearly screening vs. current practice with a 2-yearly recommendation**. *Notes: *(1) When cytology test characteristics in 2 and 3-yearly screening scenarios were varied, the direction of the incremental effect on 3-yearly screening was different for the two different 3-yearly screening scenarios. Similarly, (2) and (3): changing assumptions about CIN 3 progression to invasive cancer and early re-screening had effects of opposite directions on the two 3-yearly recommendations.

**Figure 6:**
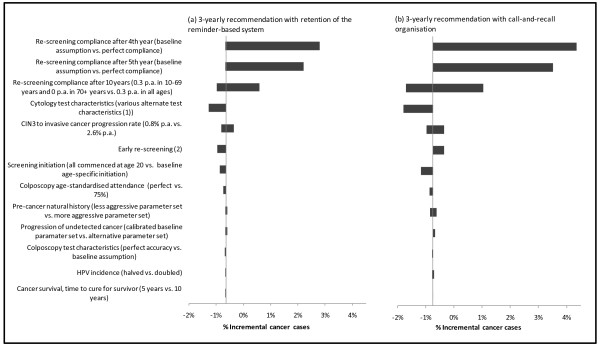
**Sensitivity analysis for incremental numbers of cervical cancer cases diagnosed with 3-yearly screening vs. current practice with a 2-yearly recommendation**. *Notes: *(1) When cytology test characteristics in 2 and 3-yearly screening scenarios were varied, the direction of the incremental effect on 3-yearly screening was different for the two different 3-yearly screening scenarios. (2) Assumptions about early re-screening had effects of opposite directions on the two 3-yearly recommendations.

Figure 4, 5, 6 summarise the findings of sensitivity analysis on the predicted incremental costs (Figure 4) and effects for the 3-yearly screening strategies, compared to current practice for 2-yearly screening. Effects are presented in two ways - in terms of life years saved or lost (Figure 5) and in terms of the relative impact on the number of cervical cancer cases diagnosed (Figure 6). Given no changes to survival assumptions, the number of cancer cases is related to the number of cervical cancer deaths, which in turn is directly related to life years lost. However, as shown in Figure 5 and 6, the relative ordering of the most influential parameters in the sensitivity analysis can vary somewhat depending on the outcome measure used.

In general, the predicted costs and effects were most sensitive to changes to assumptions about re-screening behaviour in women who were still not re-screened after 4 years. Because of the counter-intuitive baseline finding that 3-yearly screening had slightly better outcomes than 2-yearly screening, we performed extensive sensitivity analysis involving changing the assumptions about re-screening practices after 4 years of follow-up; and we found that the effect could be reversed (i.e. 3-yearly screening became slightly less effective than 2-yearly screening). However even under the most extreme assumption, in which all women not screened by 4 years in any strategy were assumed to attend in the fourth year, the effectiveness outcomes for 3-yearly screening were similar to those for 2-yearly screening, with the decrement in average life years in the population associated with 3-yearly screening predicted to be less than 0.001% (Figure 5); corresponding to an increase in the number of predicted cervical cancer cases by up to a maximum of ~4% under the most extreme assumption (Figure 6).

The costs and effectiveness findings were also somewhat sensitive to assumptions about CIN natural history, including the progression rate from CIN3 to invasive cervical cancer. The base value for the progression of CIN3 to invasive cancer in the model is age-specific (with an age-standardised value of 1.3% per annum for CIN 3 to invasive cancer); this was set after calibration to produce appropriate outcomes for age-specific rates of screen-detected high grade lesions, and invasive cancer incidence in screened and unscreened populations [8,9]. The outcomes were also somewhat sensitive to the assumed HPV incidence, and we assessed the effect of halving HPV incidence at all ages. A decrease of this magnitude is expected to occur within a few years of implementing the National HPV Vaccination Program [10] (although in practice the decrease will be concentrated mainly in younger women rather than immediately observed at all ages). In this situation the magnitude of downstream cost savings related to screening, diagnosis and treatment was similar to the baseline situation (in the baseline evaluation 3-yearly screening was associated with a 6-11% reduction in costs, whereas when HPV incidence is halved the cost reductions ranged from ~4-12%). This implies that the relative cost savings achieved by changing the screening program would still apply over the medium and longer term, taking into account changes of the magnitude induced by the effect of the National HPV Vaccination Program on HPV incidence in Australia.

In general, although estimates of absolute resource utilisation, health outcomes and costs varied with parameter variation in sensitivity analysis, the relative differences between scenarios with 2 and 3-yearly screening were robust to parameter variation. Under a wide range of parameter variations, cost savings were achieved and the predicted relative effectiveness of 2 vs. 3-yearly screening was close-to-equivalent.

## Discussion

This evaluation has found that when realistic levels of current and future screening compliance are taken into account, moving to a 3-yearly interval in Australia is not predicted to have a substantial impact on cervical cancer incidence and mortality. This finding accords with a previous evaluation of trends in cervical cancer incidence and mortality in relation to the effect of 2-yearly screening in Australia and 3-yearly screening in England; [3] and also with a large body of global evidence that suggests that the incremental value of performing screening more frequently than every 3 years (or more than every 5 years in women over 50 years of age) is limited [14,15]. After consideration of the worldwide evidence, in 2005 the International Agency for Research on Cancer recommended that screening be conducted 3-yearly in women aged 25-49 years, and 5-yearly in women aged 50-64 years [2].

A second major finding of this evaluation is that the implementation of 3-yearly screening is expected to reduce the number of diagnostic examinations and treatments conducted within the screening program, and this effect would be maximised under a call-and-recall system because of likely increased compliance to the new interval. Taken together with our finding that cervical cancer rates are not predicted to substantially change in context of 3-yearly screening, this implies that 3-yearly screening would be associated with a greater efficiency in referring the appropriate women for diagnostic evaluation. The reductions predicted in the number of treatments for high grade precancer is an important benefit associated with increasing the recommended screening interval, especially in women of reproductive age. This is because such treatments are associated with an increased risk of obstetric complications, including pre-term delivery, low birth weight and premature rupture of the membranes [16] and for some treatment modalities, increased perinatal mortality and other serious pregnancy outcomes [17]. Our baseline findings predict that 300-600 fewer women in Australia would undergo such treatments each year under a 3-yearly recommendation.

A substantial body of evidence has now found that screening in women younger than 25 years of age does not substantially lower the risk of developing invasive cervical cancer, and this evidence has been critical in informing the IARC recommendations for screening [2]. We have previously proposed that a change to the recommended age of starting screening to age 25 years could be evaluated in Australia [6]. In the current evaluation, we have not explicitly considered the impact of raising the age of starting screening, but it will be a focus of future work, particularly in the context of HPV vaccination and of potential changes to the primary screening technology. It should be noted that organizational aspects of screening are also expected to be important in such an evaluation; since the timing of screening initiation is likely to depend on whether women are specifically invited to attend for screening at age 25 years.

Our analysis has several limitations. Firstly, we did not explicitly model the impact of HPV vaccination on the risk of disease in younger cohorts of females. However, we did assess the effect of reduced HPV incidence in sensitivity analysis, and as discussed, from a safety perspective the current evaluation provides a "worst case" outcome since the overall risks of cervical cancer in the population will eventually reduce in the context of HPV vaccination. Future work will explicitly evaluate the cost-effectiveness of changes to the screening program in the context of vaccination, using multiple cohort modelling methods. Secondly, we did not include any overhead costs involved in setting up call-and-recall screening, and if this organisational change were to be made this overhead cost would need to be considered.

A further limitation of the study is the use of historical data to inform assumptions about future screening compliance. We analysed screening and management compliance over periods of 10 years after the last test, which required that we use data on 10 or more years of follow-up. However, some aspects of screening behaviour are changing over time in Australia - for example, recent data from the National Cervical Screening Program indicates that rates of early re-screening before 21 months are reducing and 5-yearly coverage rates are increasing [18]. However, our evaluation demonstrates that early re-screening has little impact on the effectiveness of screening, and the evaluation of UK re-screening behaviour demonstrates that high rates of 5-yearly coverage (for women not re-screened by 3 years) increases the overall effectiveness of screening. Therefore, the recent trends in the behaviour of Australian women should have a neutral or positive effect for the effectiveness of screening; again, this implies that our analysis provides a "worst case" estimate of outcomes from a safety perspective. Another issue is that it is unlikely that the screening compliance in Australia under new 3-yearly recommendations would precisely mirror that in other countries, because it is likely that there are other aspects of screening organisation and/or geographic issues that would influence screening compliance in Australian context. Therefore, our findings should be seen as indicative of potential outcomes in context of a move to 3-yearly screening, where the actual outcomes would be conditional on behaviour in relation to screening compliance.

Our evaluation predicts a reduction in the number of treatments for high grade lesions conducted when 3-yearly screening is recommended. This is likely to be due to the longer screening interval allowing a greater fraction of high grade cases to regress naturally, whilst maintaining a similar detection rate for those high grade lesions that would have progressed to invasive cervical cancer. Our prediction of a reduced number of high grade treatments in the National Cervical Screening Program is broadly consistent with the findings of a prior analysis of NSW registry data, which predicted a statistically non-significant decrease in histologically-confirmed high grade cases, and a significant decrease in rates of high grade cytology, in the context of 3-yearly screening [19]. However, the estimates from this prior study are not directly comparable to the current evaluation because we considered the overall effects of lifetime screening behaviour in the entire population; whereas the prior study estimated the increased risk of a high grade according to screening interval in women with 2 or more cytology tests (conditional on the first test having a negative result), and applied these estimates by pro-rating the 2-yearly participation rate to a 3-yearly situation. Our overall findings are also broadly consistent with those of another previous modelled evaluation which examined the effect of changing screening intervals to 3 years in Australia [20]. Unlike our study, this evaluation did find that 3-yearly screening would be associated with a small decrease (<5%) in the total number of life years saved by the cervical screening program; but this finding was made in context of assumed current and future perfect compliance with screening recommendations [20].

To our knowledge, the current evaluation of cervical screening in Australia is the first conducted in any setting which takes into account realistic levels of screening compliance in relation to the method of screening organisation. By harnessing detailed screening program data from three countries, we have been able to apply information from other screening programs to inform an assessment of future screening options in Australia. A move to 3-yearly screening is predicted to reduce the annual numbers of screening tests, diagnostic investigations and treatments for high grade precancers, without resulting in a substantial increase in incident cervical cancers or cervical cancer deaths. Various aspects of the National Cervical Screening Program in Australia could be re-considered over the next few years as a consequence of several factors, including the implementation of HPV vaccination, the availability of new cytology screening technologies and the potential role of primary HPV DNA testing for cervical screening. The current evaluation provides a basis for future assessments, which will also involve consideration of further extensions to the screening interval in the context of primary HPV testing.

## Conclusion

This evaluation suggests that the recommended screening interval can be safely extended from 2 to 3 years in Australia. This finding accords with the worldwide evidence on the optimal screening interval for cervical cancer screening with cervical cytology. We have quantified in detail the implications for the National Cervical Screening Program in Australia; giving predicted outcomes in context of retention of the current reminder-based system of screening organisation, or in context of a move to call-and-recall screening. In either case, a recommended 3-yearly interval is predicted to reduce the burden of diagnostic tests and treatments for high grade precancer, without resulting in a substantial increase in cervical cancer cases or deaths.

## Competing interests

The authors have no competing interests to declare. This study was funded by the National Health and Medical Research Council Australia (NHMRC Project Grant #440200) and by Cancer Council NSW. Model development was also partially funded by the Medical Services Advisory Committee, Department of Health and Ageing, Australia. The funding sources had no involvement in study design, analysis, or interpretation of results, writing of the manuscript or the decision to submit for publication.

## Authors' contributions

PC performed the modelling analysis for this evaluation, participated in the analysis of screening registry data, prepared the tables and figures and participated in drafting the manuscript. JBL led the development of the Australian screening model used in this evaluation, checked and repeated the analysis, and performed sensitivity analysis. MC led the analysis of screening registry data and conceived the basic design of the screening model. MS participated in data analysis and model development and was responsible for the collation and integration of epidemiological data into the model. KH prepared cost information for the model and guided the interpretation of cost-effectiveness outcomes. SD and SL performed systematic review of cytology accuracy for the model and assisted in the specification of model screening and management pathways. KC conceived and led the project, participated in all aspects of the analysis, and drafted the manuscript. All authors read and approved the final manuscript.

## Authors' Information

Prudence Creighton BSc, Research Programmer, Cancer Epidemiology Research Unit, Cancer Council New South Wales, Sydney, Australia. (Current address: School of Public Health and Community Medicine, University of NSW, Sydney, Australia)

Jie Bin Lew BSc, Senior Research Programmer, Cancer Epidemiology Research Unit, Cancer Council New South Wales, Sydney, Australia.

Mark Clements PhD, Research Fellow, National Centre for Epidemiology and Population Health, Australian National University, Canberra, Australia.

Megan Smith BE MPH, Cervical Modelling Program Manager, Cancer Epidemiology Research Unit, Cancer Council New South Wales, Sydney, Australia.

Kirsten Howard PhD, Senior Lecturer, School of Public Health, The University of Sydney, Sydney, Australia.

Suzanne Dyer PhD, Systematic Reviews and Health Care Assessment, NHMRC Clinical Trials Centre, The University of Sydney, Sydney, Australia.

Sarah Lord MBBS MS(Epi), Epidemiologist, NHMRC Clinical Trials Centre & The Screening and Test Evaluation Program, The University of Sydney, Sydney, Australia.

Karen Canfell DPhil, Sydney Rotary Research Fellow, Cancer Epidemiology Research Unit, Cancer Council New South Wales, Sydney, Australia.

## Pre-publication history

The pre-publication history for this paper can be accessed here:

http://www.biomedcentral.com/1471-2458/10/734/prepub
